# Model consent clauses for rare disease research

**DOI:** 10.1186/s12910-019-0390-x

**Published:** 2019-08-01

**Authors:** Minh Thu Nguyen, Jack Goldblatt, Rosario Isasi, Marlene Jagut, Anneliene Hechtelt Jonker, Petra Kaufmann, Laetitia Ouillade, Fruszina Molnar-Gabor, Mahsa Shabani, Eric Sid, Anne Marie Tassé, Durhane Wong-Rieger, Bartha Maria Knoppers, on behalf of the IRDiRC-GA4GH Model Consent Clauses Task Force, Jack Goldblatt, Rosario Isasi, Petra Kaufmann, Bartha Knoppers, Fruzsina Molnar Gabor, Minh Thu Nguyen, Laetitia Ouillade, Eric Sid, Masha Shabani, Anne-Marie Tassé, Durhane Wong-Rieger

**Affiliations:** 1 Center of Genomics and Policy, McGill University, Montreal, Quebec H3A 0G1 Canada; 2 University of Western Australia, Perth, Australia; 3 Institute for Bioethics and Health Policy, University of Miami, Miami, USA; 4 IRDiRC Scientific Secretariat, Inserm US-14, Paris, France; 5 AveXis, Chicago, USA; 6 AFM-Téléthon, Evry, France; 7 Heidelberg Academy of Sciences and Humanities, Heidelberg, Germany; 8 Centre for Biomedical Ethics and Law, Brussels, Belgium; 9 National Center for Advancing Translational Sciences, National Institutes of Health, Bethesda, USA; 10 Canadian Organization for Rare Disorders, Toronto, Canada

**Keywords:** Rare diseases, Informed consent, Research ethics, Core consent elements, Consent clauses

## Abstract

**Background:**

Rare Disease research has seen tremendous advancements over the last decades, with the development of new technologies, various global collaborative efforts and improved data sharing. To maximize the impact of and to further build on these developments, there is a need for model consent clauses for rare diseases research, in order to improve data interoperability, to meet the informational needs of participants, and to ensure proper ethical and legal use of data sources and participants’ overall protection.

**Methods:**

A global Task Force was set up to develop model consent clauses specific to rare diseases research, that are comprehensive, harmonized, readily accessible, and internationally applicable, facilitating the recruitment and consent of rare disease research participants around the world. Existing consent forms and notices of consent were analyzed and classified under different consent themes, which were used as background to develop the model consent clauses.

**Results:**

The IRDiRC-GA4GH MCC Task Force met in September 2018, to discuss and design model consent clauses. Based on analyzed consent forms, they listed generic core elements and designed the following rare disease research specific core elements; Rare Disease Research Introductory Clause, Familial Participation, Audio/Visual Imaging, Collecting, storing, sharing of rare disease data, Recontact for matching, Data Linkage, Return of Results to Family Members, Incapacity/Death, and Benefits.

**Conclusion:**

The model consent clauses presented in this article have been drafted to highlight consent elements that bear in mind the trends in rare disease research, while providing a tool to help foster harmonization and collaborative efforts.

**Electronic supplementary material:**

The online version of this article (10.1186/s12910-019-0390-x) contains supplementary material, which is available to authorized users.

## Background

Over the last decade, rare disease research has seen tremendous developments in diagnostic efficiency and therapeutic interventions [1]. Contributing factors include the discovery of novel genes with new sequencing technologies, global collaborative efforts and commitments, and improved data and resource sharing. Adding to this are the growing number of genotype-phenotype datasets and matchmaking platforms, along with the trend towards patient-centered research, whereby patient organizations play a leading role in data generation and research recruitment. To maximize the impact of these initiatives in contributing substantive amounts of quality data for research use, practical model consent clauses are essential to enhance data interoperability as well as to meet the informational needs of participants, ensure proper ethical and legal use of data sources and participants’ overall protection.

To address this need, the International Rare Diseases Research Consortium (IRDiRC) and the Global Alliance for Genomics and Health (GA4GH) met to develop model consent clauses for rare disease research. The following proposed consent clauses are guided by the *Framework for Responsible Sharing of Genomics and Health-Related Data* [2] and other international and national ethics and legal consent frameworks. They provide the foundation for the harmonization and standardization of participant recruitment and consent processes for rare disease research. Although not all the proposed clauses are exclusive to the rare disease context, certain clauses are of particular importance to consent procedures for rare disease research and complement existing core elements found in “classical” or “generic” consent forms. It is hoped that together these clauses will ensure that research involving patients with rare conditions be deployed effectively to promote and catalyze collaborative multinational studies through interoperable and responsible research practices endorsed by IRDiRC and GA4GH.

### Consent issues in rare disease research

Obtaining informed consent from research participants not only respects personal autonomy, self-determination, and the right to privacy but also seeks to prevent undue harm [3–5]. Research participants must be well informed of the research goals, benefits and risks, and the possibility to refuse participation and withdraw from research at any time without affecting their medical care. The voluntary expression of consent is fundamental to ethical research practices. In the rare disease research context, however, consent processes have become complex in the current landscape of technological and genomic advances, along with the extensive collection, pooling and dissemination of data worldwide. Because of the scarcity of patients and the need to share information internationally to find similar cases, data sharing and ‘matchmaking’ is imperative for rare disease research. Since most rare diseases appear in childhood, the recruitment of children and unaffected family members might further complicate consent processes [6]. Particular phenotypes of rare disease patients also often require the collection and sharing of audiovisual data (e.g., facial images, videos, etc.), the use of machine learning procedures for data phenotyping [7], and the bridging between clinical care and research [8] in this scientific domain.

Notably, the challenge in establishing consent policies for rare disease research stems from the dichotomy between the push for free-flow of data against concerns about loss of privacy. Patients with rare diseases often expect that data are shared for scientific advances in genomic medicine and rare diseases research. At the same time, patients are concerned about being identified, a risk inherent to data sharing [9] and enhanced in the rare disease context. Particularly, the likelihood of individual re-identification from genomic data, whether coded or anonymized, has been documented [10, 11], especially when such data has been linked with other sensitive familial, sociodemographic or audiovisual information. This is why international policy approaches to assess privacy risks in genomic research widely adopt as a criterion the “reasonable likelihood test” (i.e., based on the proportionate evaluation of real risks and benefits and the balance of probabilities, the possible benefits for participants must surpass potential consequences for their privacy) [12].

The singularity and diversity of rare diseases, combined with the small number of patients for each disorder, effectively precludes conventional research discovery approaches, including those directed at addressing privacy risks and concerns. Some rare disease research mandates cross-matching data between different centers for discovery and diagnostic purposes. Thus, absolute privacy protection is unrealistic in this realm. Coding and security tools serve to mitigate and protect against privacy risks. However, re-identification is often desired by patients for the return of results and so precludes anonymization [7, 13].

Hence, research involving rare disease participants is challenged by unique realities and overall impediments to genomic research, and thus, should be acknowledged in ethical and legal deliberations regarding research protocols, privacy protections, and consent standards. Rare disease participants are often well informed about their disease, highly motivated to participate in different research studies, and strong advocates for greater access to research. They may view identifiability risks as minor when weighed against the opportunity to gain a diagnosis or to support research advancements towards new therapies. Consequently, privacy interpretations should be broadened to include not only the right to confidentiality, secrecy and non-interference but also the positive right to “determine and manage personal information, and to actively have a say in one’s own private sphere” [14] and to realize the human right of everyone to “share in scientific advancements and its benefits [15, 16].” Moreover, privacy protections should go beyond rare disease participants to also protect and further the interests of their family members in familial and trio genomic studies [17].

As such, the adoption of governance frameworks, security measures, and standards (i.e., data management/access policies, Privacy Preserving Record Linkage [18] or unique identifying systems) has become the nexus from which privacy discourse has shifted [19]. Adding to this is a move towards a nuanced approach to consent standards that account for the unique complexities and specificities of rare disease research [20]. By obtaining proper informed consent, proportionality between protecting the rights and interests of rare disease participants and promoting good research is achievable. In fact, qualitative studies show that knowledgeable rare disease patients understand the need for large-scale data sharing and expect their data to be distributed and reused but require, nonetheless, that they be informed of such activities in order to maintain a level of protection and control [21, 22].

## Methods

In January 2018, a joint IRDiRC and GA4GH Model Consent Clauses (MCC) Task Force was charged to develop model consent clauses relevant to rare disease research. Both IRDiRC and GA4GH asked their members to nominate members to the Task Force, and the Chairs of the Task Force decided on the final composition of the Task Force. The Task Force was composed of 15 members from 8 different countries, covering different expertise areas including law, ethics, health policy, research and clinical experience. Two members were representatives of patient organizations, e.g., Canadian Organization for Rare Disorders (CORD) and the French Muscular Dystrophy Association (AFM-Téléthon). The objective of the Task Force was to create consent clauses that are comprehensive, harmonized, readily accessible, and internationally applicable, facilitating the recruitment and consent of rare disease research participants around the world. The Scientific Secretariat of IRDiRC first reached out to all IRDiRC Consortium Assembly members and all Task Force members with the following question:

*“In your work/experience or in the 'best case scenario,' are there clauses you have used in rare disease research consent forms that are not usually found or should be found on generic biomedical research forms?*”

Five IRDiRC Consortium Assembly members and three Task Force members were able to share their consent forms and clauses, contributing to a total of 35 consent forms and/or notice of consent. Additionally, 5 publications from the Clinical Sequencing Evidence-Generating Research (CSER) and Electronic Medical Records and Genomics (eMERGE) projects were analyzed [23–27]. The clauses were classified under different consent themes and were used as references.

## Results

In September 2018, the MCC Task Force met at the Rare Disease Platform in Paris, France, to develop and draft the model consent clauses (Additional file 1). The meeting consisted of background presentations, iterating the role and objectives of IRDiRC and GA4GH, as well as provision of a historical overview of the evolution of consent forms used in rare disease research over the past 8–10 years, along with emerging trends (see Table 1). The group identified different research themes that are specific and crucial for rare disease research. Based on a compilation of already existing consent form language, the experts then developed *Generic and Rare Disease Specific Core Consent Elements* (Table 2) and *Model Consent Clauses for Rare Disease Research* (Table 3) that take into account international socio-ethical, legal and cultural differences as well as keeping the patients’ perspectives in mind.

**Table 1:** Emerging Trends in Rare Disease Research with Consent Implications

1. Increased complexity of research methods	
• Researchers are increasingly exploring new approaches to identify rare genetic variants resulting in increased complexity and diversity of research methods and protocols;	
• Clinical care often involves research in the context of specialty clinics providing care for rare disease patients.	
Consent implications:	
• Consent forms risk becoming lengthy, technical and complicated;	
• The study purpose and potential benefits must be clearly stated to manage participant expectations and provide them with the necessary information to make informed decisions.	
2. Increased family enrollment as a unit	
• Family members can provide important information in identifying the cause of a rare genetic disease (increased diagnostic rate – singleton vs. trio analysis) or help identify de novo mutations in a family;	
• Establishment of family pedigrees to allow linkage of family data.	
Consent implications:	
• Although each family member may undergo individual consent interviews, often families are recruited using one consent document to address ethical and administrative challenges (i.e., no distinction made between “affected” and “unaffected” family members in consent forms);	
• Disclose privacy protections for use and sharing of family data;	
• Address possible coercion or undue inducement from family members participating in research study;	
• Address the return of results to family members;	
• Provide the possibility to notify family members of research results in case of the participant’s incapacity or death.	
3. Increased data collection (amount, type & frequency)	
• Types of data collected: medical (e.g., clinical test results, diagnoses), health (e.g., administrative, self-reported data, sociodemographic), family history, genetic and phenotypic data;	
• Data sources: hospitals, private clinics, research, registries, social network sites;	
• Ongoing data acquisition from medical records.	
Consent implications:	
• Disclose information, privacy and identifiability risks to participants and their family members.	
4. Increased use of audiovisual data	
• 2–3 dimensional facial imaging used to identify and analyze patterns and similarities associated with facial dysmorphology;	
• Effective in identifying the underlying cause of rare diseases with computational phenotyping.	
Consent implications:	
• Disclose information, privacy and identifiability risks to participants.	
5. Increased global data sharing and linkage	
• Global data sharing, data linkage and international collaboration allow achieving sample sizes of statistical significance, richer datasets and ability to “match” similar genotypes/phenotypes for gene discovery in rare diseases;	
• Data linkage minimizes the burden on participants to submit new or additional data;	
• Large-scale data sharing and linkage increases complexity of data management and handling (e.g. access policies, security measures, data coordination, use of unique personal identifiers).	
Consent implications:	
• Disclose information, privacy and identifiability risks to participants;	
• Inform participants of sharing process and limited right to withdraw data.

**Table 2:** Generic and Rare Disease Research Specific Core Consent Elements

Generic Core Elements:	
General information/Introduction (name of researchers, hospital/institution, funders/sponsors, etc.)	
Nature and objectives of the study	
Voluntariness of participation	
Procedures involved in participation (what will happen during the study) /types of data and samples that will be collected	
Possibility of large scale genome-wide sequencing techniques	
Potential physical, psychological, social and informational risks	
Potential benefits of participation	
Protections in place [locally] to ensure security/privacy/confidentiality	
Duration/place of data/sample storage	
Hosting of data in an open access database	
Access to data/samples for research purposes (who will have access, types of access, governance framework, procedures in place – ex. data access committee), including access by pharma/industry, if applicable	
Access to data/samples for purposes of auditing, validation, control, etc.	
Return of research results/incidental findings (processes and potential inclusion in medical records)	
Withdrawal procedures (sample/data retrieval, destruction, no further contact, no further access, unlink, no further use, etc.)	
Compensation/reimbursement	
Prospects for commercialization and intellectual property procedures	
Study dissemination/publication	
Assent (where applicable)	
Re-contact	
Study oversight (IRB/REC/REB)	
Core Elements (Rare Disease Research Specific):	
Rare Disease Research Introductory Clause	
Familial Participation	
Audio/Visual Imaging	
Collecting, storing, sharing of rare disease data	
Recontact for matching	
Data Linkage	
Return of Results to Family Members	
Incapacity/Death	
Risks and Benefits

**Table 3:** Model Consent Clauses for Rare Disease Research

	Consent Elements:	Sample Clauses:
1	Rare Disease Research Introductory Clause	*The consent information below contains special features unique to rare disease research. There is often a need to: use audiovisual images; involve family members; share your information (inter-)nationally with clinicians and researchers; create reference databases; and look for those who are similar. Registries and databases will be created to put all participants and their families together so as to better understand your condition.*
2	Familial Participation	*Information about your family history will be collected and used to interpret your results and build a family tree (also called a “pedigree”). Creating a family tree will allow us to link or connect your personal health data with those of your family members.*
		*If needed, fluid or tissue samples and relevant medical history may be taken from consented family members, such as your parents or siblings.*
3	Audio/Visual Imaging	*Audio/ images may be taken, if necessary, as certain physical features may be associated with specific conditions. It may also be helpful to use audiovisual imaging already taken [*e.g. *from your medical records and/or registries]. Often with rare disease research, the results will be used in teaching and publications. Efforts will be taken to protect your privacy, however, the risk remains that you might be identified.*
4	Collecting, storing, sharing of data	*Your collected data will be stored in databases or registries that meet security and safety standards.*
		*Rare disease research often requires access to and the creation of large datasets. Therefore, your data will be shared with researchers and research databases after ethics approval. Such research can take place in universities, hospitals, non-profit groups, companies, and/or government laboratories.*
		*Storing and sharing of data help to find people with the same condition or with similar clinical features (a process also known as “matching”). This may also help to better understand your condition and to facilitate recruitment for clinical trials, develop new tools, and improve diagnostics and therapies.*
5	Recontact for matching	*Since this study includes matching, if a potential match is found, you will be notified.*
		[add opt-in/out option if needed]
6	Data Linkage	*In order to improve data completeness and to help interpret your data, we need to link your data from different sources (*e.g. *medical files, administrative health databases, registries, data from family members,* etc.*).*
7	Return of Results to Family Members	*It takes a long time to interpret research data accurately. Reports on research progress as well as general results will be made available* via *[…..].*
		*Please indicate below if you wish to be notified about individual results specific to you about the rare disease in your family.*
		[add opt-in/out option if needed]
		*If you have indicated that you wish to be notified about individual results, you will be contacted by […].*
		[Insert any time limitations affecting this notification].
		*In addition,* [insert local return policy if necessary]
		*Due to the limitations of resources, your data may not be re-analyzed in the future.*
8	Incapacity/Death	*Your data are important for family members and for research on your condition. Therefore, your data will be kept and used even if you become incapacitated or die.*
		*If you become incapacitated or die, you can name a person to be notified of results that are relevant to the health of your family members.*
		[Yes (Insert name of person to be notified) / No]
9	Benefits	*You may or may not benefit from participating in this study. In rare disease research, because of the small sample size, the quality of the research and the possible benefits to you and your family and others with similar conditions requires: involving families; creating large datasets; sharing, linking and matching data; and using audio visual images.*

The model consent clauses presented below serve as a practical tool to assist in the development of consent forms and research protocols. These clauses aim to provide examples of consent categories that are specific to rare disease research and therefore do not cover all necessary components of a standard consent form (see: Table 2*: Generic and Core Consent Elements for Rare Disease Research*). Local laws, regional policy and guidelines may extend or modify these consent requirements and therefore variation can be expected.

In addition to the introductory consent clauses regarding the purpose of the research, name of researchers/sponsors etc., rare disease consent forms could use introductory clauses to draw attention to some of the unique features that distinguish it from the standard core consent elements. These clauses must be customized to each project’s specific needs.

## Discussion

Scientific breakthroughs in research and clinical fields have brought immense opportunities for potential diagnostic tools and therapies to those affected by a rare disease. Since the completion of the Human Genome Project [28], much progress has been made regarding technological advances, the growth of increased access to genomic sequencing, and the translation of gene-editing techniques into the first in-human trials [29]. These developments have raised the hope for treatments for rare and genetic disorders previously considered incurable. As the prospects of these and other new technologies continue to evolve, many patients expect that researchers share data to accelerate the path towards a diagnosis and ultimately towards effective treatments. For many patients, the benefit of research advances may come too late if progress is not accelerated through sharing resources and data. Researchers and ethics review boards therefore need to take into account the socio-ethical and legal challenges that these developments will present for recruitment and consent processes. Overly-rigid interpretations of consent will be counterproductive in the context of rare disease, to say nothing of the fact that current consent forms have generally been criticized for being incomprehensible to research participants.

## Conclusion

The model consent clauses presented above have been drafted to highlight consent elements that bear in mind the trends in rare disease research, and genomic research in general, while providing a tool to foster harmonization and collaborative efforts.

### Limitations

The MCC Task Force recognizes the need for further guidance regarding consent procedures for the recruitment of minors (e.g., assent and re-contact at age of majority clauses) and the nuances pertaining to the communication of results to family members and between family members. These aspects were beyond the scope of the workshop.

## Additional file

Additional file 1: MCC Report of the Model Consent Clauses Task Force Meeting (Paris, September 6–7, 2018). (DOCX 138 kb)

## Data Availability

Not applicable.

## Abbreviations

CSER: Clinical Sequencing Evidence-Generating Research
eMERGE: Electronic Medical Records and Genomics
GA4GH: Global Alliance for Health and Genomics
IRB: Institutional Review Board
IRDiRC: International Rare Diseases Research Consortium
MCC: Model Consent Clauses
REB: Research Ethics Board
REC: Research Ethics Committee

## References

[1] Dawkins HJS, Draghia-Akli R, Lasko P, Lau LPL, Jonker AH, Cutillo CM (2018). Progress in rare diseases research 2010-2016: an IRDiRC perspective. Clin Tranl Sci.

[2] Global Alliance for Genomics and Health, “Framework for responsible sharing of genomics and health-related data; c2014. Available from: https://www.ga4gh.org/genomic-data-toolkit/regulatory-ethics-toolkit/framework-for-responsible-sharing-of-genomic-and-health-related-data/. [cited 2018 Dec 19]

[3] World Medical Association (2013). World medical association declaration of Helsinki: ethical principles for medical research involving human subjects. JAMA..

[4] World Medical Association. World Medical Association Declaration of Taipei on Ethical Considerations regarding Health Databases and Biobanks. Available from: https://www.wma.net/policies-post/wma-declaration-of-taipei-on-ethical-considerations-regarding-health-databases-and-biobanks/. [cited 2018 Dec 19]

[5] World Medical Association. World Medical Association Statement on Genetics and Medicine. Available from: https://www.wma.net/policies-post/wma-statement-on-genetics-and-medicine/. [cited 2018 Dec 19]

[6] Rahimzadeh V, Schickhardt C, Knoppers BM, Sénécal K, Vears DF, Fernandez CV (2018). Key implications of data sharing in pediatric genomics. JAMA Pediatr.

[7] Hallowell Nina, Parker Michael, Nellåker Christoffer (2018). Big data phenotyping in rare diseases: some ethical issues. Genetics in Medicine.

[8] Bertier G, Cambon-Thomsen A, Joly Y (2018). Is it research or is it clinical? Revisiting an old frontier through the lens of next-generation sequencing technologies. Eur J Med Genet.

[9] Heeney C, Hawkins N, de Vries J, Boddington P, Kaye J (2011). Assessing the privacy risks of data sharing in genomics. Public Health Genomics.

[10] Kaye J (2012). The tension between data sharing and the protection of privacy in genomics research. Annu Rev Genomics Hum Genet.

[11] Gutmann A (2013). Data re-identification: prioritize privacy. Science..

[12] Global Alliance for Genomics and Health: Privacy and Security Policy, 2015. Available from: https://www.ga4gh.org/wp-content/uploads/Privacy-and-Security-Policy.pdf. [cited 2018 Dec 19].

[13] Hansson MG, Lochmüller H, Riess O, Schaefer F, Orth M, Rubinstein Y (2016). The risk of re-identification versus the need to identify individuals in rare disease research. Eur J Hum Genet.

[14] Mascalzoni D, Paradiso A, Hansson M (2014). Rare disease research: breaking the privacy barrier. Appl Transl Genomics.

[15] UN General Assembly (1948). Universal declaration of human rights.

[16] United Nations (1966). International covenant on economic, social and cultural rights.

[17] Takashima K, Maru Y, Mori S, Mano H, Noda T, Muto K (2018). Ethical concerns on sharing genomic data including patients’ family members. BMC Med Ethics.

[18] Baker D, Knoppers BM, Phillips M, van ED, Kaufmann P, Lochmuller H, et al. Privacy-preserving linkage of genomic and clinical data sets. IEEE/ACM Trans Comput Biol Bioinform. 2018:1–1. 10.1109/TCBB.2018.2855125. 10.1109/TCBB.2018.2855125 30059313

[19] Knoppers BM, Chadwick R (2015). The ethics weathervane. BMC Med Ethics..

[20] Gainotti S, Turner C, Woods S, Kole A, McCormack P, Lochmüller H (2016). Improving the informed consent process in international collaborative rare disease research: effective consent for effective research. Eur J Hum Genet.

[21] McCormack P, Kole A, Gainotti S, Mascalzoni D, Molster C, Lochmüller H (2016). ‘You should at least ask’. The expectations, hopes and fears of rare disease patients on large-scale data and biomaterial sharing for genomics research. Eur J Hum Genet.

[22] Darquy S, Moutel G, Lapointe A-S, D’Audiffret D, Champagnat J, Guerroui S (2016). Patient/family views on data sharing in rare diseases: study in the European LeukoTreat project. Eur J Hum Genet.

[23] Henderson GE, Wolf SM, Kuczynski KJ, Joffe S, Sharp RR, Parsons DW (2014). The challenge of informed consent and return of results in translational genomics: empirical analysis and recommendations. J Law Med Ethics J Am Soc Law Med Ethics.

[24] Tomlinson AN, Skinner D, Perry DL, Scollon SR, Roche MI, Bernhardt BA (2016). “Not tied up neatly with a bow”: professionals’ challenging cases in informed consent for genomic sequencing. J Genet Couns.

[25] Bernhardt BA, Roche MI, Perry DL, Scollon SR, Tomlinson AN, Skinner D (2015). Experiences with obtaining informed consent for genomic sequencing. Am J Med Genet A.

[26] Brothers KB, Lynch JA, Aufox SA, Connolly JJ, Gelb BD, Holm IA (2014). Practical guidance on informed consent for pediatric participants in a biorepository. Mayo Clin Proc.

[27] Trinidad SB, Fullerton SM, Bares JM, Jarvik GP, Larson EB, Burke W (2012). Informed consent in genome-scale research: what do prospective participants think?. AJOB Prim Res.

[28] Collins FS, Green ED, Guttmacher AE, Guyer MS (2003). US National Human Genome Research Institute. A vision for the future of genomics research. Nature..

[29] Cyranoski D (2016). CRISPR gene-editing tested in a person for the first time. Nature..

