# Supplementary material for: Model consent clauses for rare disease research
Source: BMC Med Ethics. 2019 Aug 1;20:55. doi: 10.1186/s12910-019-0390-x (PMC6676617; doi:10.1186/s12910-019-0390-x)
Supplement: Supplementary file 1 — MCC Report of the Model Consent Clauses Task Force Meeting (Paris, September 6–7, 2018). (DOCX 138 kb) [file 12910_2019_390_MOESM1_ESM.docx]

Meeting report series

Report of the Model Consent Clauses Task Force

Paris, France

September 6-7, 2018

Participants

Jack Goldblatt, University of Western Australia, Australia (Co-Chair)

Bartha Knoppers, Center of Genomics and Policy, McGill University, Canada (Co-Chair)

Rosario Isasi, Institute for Bioethics and Health Policy, USA

Fruzsina Molnar Gabor, Heidelberg Academy of Sciences and Humanities, Germany

Minh Thu Nguyen, Center of Genomics and Policy, McGill University, Canada

Laetitia Ouillade, Atos/Telethon, France

Eric Sid, NCATS/NIH, USA

Masha Shabani, Centre for Biomedical Ethics and Law, Belgium

Anne-Marie Tassé, Center of Genomics and Policy, McGill University, Canada

Durhane Wong-Rieger, CORD, Canada

Anneliene Jonker , IRDiRC Scientific Secretariat, France

Marlène Jagut, IRDiRC Scientific Secretariat, France

Apologies

Petra Kaufmann, AveXis, USA (Co-Chair)

Jill Clayton-Smith, University of Manchester, UK

Laura Rodriguez, NHGRI/NIH, USA

Susan Wallace, University of Leicester

Agenda

September 6, 2018 – Workshop MCC day 1

| 9:00 – 9:15 | Introduction – Welcome | Anneliene Jonker |
| --- | --- | --- |
| 9:15 – 9:30 | IRDiRC | Jack Goldblatt |
| 9:30 – 9:45 | GA4GH | Bartha Knoppers |
| 9:45 – 10:30 | Rare Disease Research Consent (Issues & Challenges) | Minh Thu Nguyen |
| 10:30 – 11:00 | Coffee break |  |
| 11:00 – 12:30 | Review of rare disease consent clauses | Bartha Knoppers |
| 12:30 – 13:30 | Lunch break |  |
| 13:30 – 15:30 | Review of rare disease consent clauses | Bartha Knoppers |
| 15:30 – 16:00 | Coffee break |  |
| 16:00 – 17:00 | Review of rare disease consent clauses - continued | Bartha Knoppers |
| 17:00 | End of day 1 workshop |  |
| 19:30 | Group dinner | Au Moulin Vert, 33 rue du Moulin Vert  http://www.aumoulinvert.com |

September 7, 2018 – Workshop MCC day 2

| 9:00 – 10.30 | Finalizing consent clauses | Bartha Knoppers |
| --- | --- | --- |
| 10:30 – 11:00 | Coffee break |  |
| 11:00 – 12:30 | Outline for article | Bartha Knoppers |
| 12:30 – 14:00 | Lunch break |  |
| 14:00 | End of workshop |  |

REPORT

**Model Consent Clauses Task Force designs specific clauses for rare diseases research**

The heterogeneity of rare diseases, combined with the small number of patients for each disorder, often precludes conventional discovery approaches. To facilitate international data sharing, coordinated consent processes between research centers is crucial. While an International Framework for Data Sharing and accompanying policies have been developed by the [Global Alliance for Health (GA4GH)](http://www.ga4gh.org/), practical and procedural tools for consent elements in the context of rare diseases are lacking, in particular, model consent clauses for pediatric rare disease research. To address these challenges, the joint IRDiRC and GA4GH [Model Consent Clauses (MCC) Task Force](http://www.irdirc.org/activities/task-forces/model-consent-clauses-for-rare-disease-research-task-force/) had the objective to gather rare disease research policy experts to develop model consent clauses specific to rare diseases that are comprehensive, harmonized, readily-accessible, and internationally applicable, enabling the recruitment and consent of rare disease research participants around the world.
 
The MCC Task Force members met on September 6-7, 2018, at the Rare Diseases Platform in Paris (France). The meeting started with background presentations of both IRDiRC and the GA4GH as well as an historical analysis of the evolution of consent forms used in rare disease research over the past 8 years. The group identified the aspects of research that require consent and that are specific and crucial for rare disease research. Based on a compilation of already existing consent form language, the experts then developed model consent clauses specific to rare diseases considering international socio-ethical, legal and cultural differences as well as keeping the patient’s perspective in mind.
 
A paper is currently under development, which will lay out the state of play for consents for rare disease research, the model consent clauses designed during this workshop, and considerations regarding the impact of new diagnostics/therapeutics technologies on the consents of participants in the future. Hopefully, this resource will further benefit both IRDiRC’s and GA4GH’s aim to promote and catalyze collaborative multi-national studies through interoperable and responsible research practices.
